# Exploring the Intersection of Schizophrenia, Machine Learning, and Genomics: Scoping Review

**DOI:** 10.2196/62752

**Published:** 2024-11-15

**Authors:** Alexandre Hudon, Mélissa Beaudoin, Kingsada Phraxayavong, Stéphane Potvin, Alexandre Dumais

**Affiliations:** 1 Department of psychiatry and addictology Faculty of Medicine Université de Montréal Montréal, QC Canada; 2 Centre de recherche de l'Institut universitaire en santé mentale de Montréal Montréal, QC Canada; 3 Institut universitaire en santé mentale de Montréal Montréal, QC Canada; 4 Department of psychiatry and addictology Université de Montréal Montréal, QC Canada; 5 Faculty of Medicine McGill University Montréal, QC Canada; 6 Services et Recherches Psychiatriques AD Montréal, QC Canada; 7 Institut nationale de psychiatrie légale Philippe-Pinel Montréal, QC Canada

**Keywords:** schizophrenia, genomic data, machine learning, artificial intelligence, classification techniques, psychiatry, mental health, genomics, predictions, ML, psychiatric, synthesis, review methods, searches, scoping review, prediction models

## Abstract

**Background:**

An increasing body of literature highlights the integration of machine learning with genomic data in psychiatry, particularly for complex mental health disorders such as schizophrenia. These advanced techniques offer promising potential for uncovering various facets of these disorders. A comprehensive review of the current applications of machine learning in conjunction with genomic data within this context can significantly enhance our understanding of the current state of research and its future directions.

**Objective:**

This study aims to conduct a systematic scoping review of the use of machine learning algorithms with genomic data in the field of schizophrenia.

**Methods:**

To conduct a systematic scoping review, a search was performed in the electronic databases MEDLINE, Web of Science, PsycNet (PsycINFO), and Google Scholar from 2013 to 2024. Studies at the intersection of schizophrenia, genomic data, and machine learning were evaluated.

**Results:**

The literature search identified 2437 eligible articles after removing duplicates. Following abstract screening, 143 full-text articles were assessed, and 121 were subsequently excluded. Therefore, 21 studies were thoroughly assessed. Various machine learning algorithms were used in the identified studies, with support vector machines being the most common. The studies notably used genomic data to predict schizophrenia, identify schizophrenia features, discover drugs, classify schizophrenia amongst other mental health disorders, and predict the quality of life of patients.

**Conclusions:**

Several high-quality studies were identified. Yet, the application of machine learning with genomic data in the context of schizophrenia remains limited. Future research is essential to further evaluate the portability of these models and to explore their potential clinical applications.

## Introduction

Schizophrenia is a complex mental health disorder that can have a significant negative impact on patients’ resilience, quality of life, and self-esteem [1]. Considering the heterogenous nature of schizophrenia, several fields of research, such as genomics, also use the terminology psychotic disorder spectrum to refer to schizophrenia-like disorders [2]. Furthermore, while untreated, this mental health condition can lead to violence and violent offending [3]. A recent review of the literature estimated that schizophrenia has the highest societal cost among all mental health diseases. Indeed, reports from 10 countries estimated schizophrenia-related costs per person per year to be around US $2004-$94,229, with considerable variability amongst countries [4]. Despite several treatments being available, such as antipsychotics (dopamine receptor antagonists and partial agonists), up to 20%-30% of patients will remain treatment-resistant, and further approaches, such as cognitive behavioral therapy, will be used as adjuncts [5-7]. Various studies have explored the diverging clinical presentations of patients with schizophrenia and developed complexity estimators to aid clinicians in understanding the neuropathological processes involved in this complex illness [8,9]. Among recent research, several key factors have been identified as being linked to the development of the disorder, such as the length of the first psychotic episode, hormonal variations, as well as the presence of negative symptoms [10]. Despite the current knowledge that early interventions can help in the prognosis of patients diagnosed with schizophrenia, no prediction model is used in clinical practice as they usually do not account for variance between individuals [11].

To account for this variance and the dimensional aspects of schizophrenia, there have been tremendous efforts to gather genomic data and in-depth knowledge of neurobiological aspects of this disorder [12]. The entirety of the genetic information contained in an organism’s DNA is referred to as genomic data [13-15]. This comprises details on gene structure, function, and variation in addition to the nucleotide sequence (adenine, thymine, cytosine, and guanine) found in the genome [16]. Genomic data is used to research the genetic contributions to traits, diseases, and biological processes [17]. It includes a variety of genetic information, such as single nucleotide polymorphisms (SNPs), copy number variations (CNVs), and gene expression patterns [18]. Worldwide collaborations have resulted in genome-wide association studies (GWAS) in over 56,000 schizophrenia cases and 78,000 controls, which identified 270 distinct genetic loci and polygenic risk scores, which can currently explain around 7.7% of the variance in schizophrenia case-control status [19]. Despite over 300 studies on gene expression in schizophrenia conducted over the past 15 years, none has consistently identified specific genes that contribute to schizophrenia risk [20]. Due to the complexity of schizophrenia, novel approaches are essential to better understand its neurobiological basis and improve outcome predictions, as it involves a network of genetic, neural, behavioral, and environmental factors [21].

Among novel approaches, machine learning has been increasingly used in the latest decade for various applications in medicine [22]. Machine learning is a branch of artificial intelligence that deals with teaching computers how to learn from and make predictions or judgments based on data through the use of statistical models and algorithms [23,24]. It focuses on creating systems that, through experience, may naturally perform better on a given task without having to be specifically designed to do so [25]. Data used by machine learning algorithms are referred to as model features [26]. Recent advancements in the field of data science have demonstrated that precision and genomic medicine combined with artificial intelligence have the potential to improve patient health care [27]. Examples of such advancements are the possibility of conducting variant calling, genome annotation and variant classification, and phenotype-to-genotype correspondence by using machine learning algorithms [28]. While existing literature reviews have explored specific applications of machine learning using genomic data for schizophrenia, none, to our knowledge, have comprehensively examined the diverse uses of machine learning at the intersection of these three fields, which could enhance the understanding of schizophrenia, thereby justifying the necessity for a thorough literature review. [29,30]. By identifying the broader applications of machine learning in this context, this overview will help researchers and clinicians pinpoint gaps in current research and pave the way for future applications of machine learning in the study of schizophrenia using genomic data.

This study aims to identify the various applications of machine learning algorithms using genomic data in the field of schizophrenia. By examining these approaches, this research offers an initial exploration into the methods being investigated to address the complexities of schizophrenia, a significant yet challenging mental illness. Therefore, this scoping review aimed to provide a comprehensive overview of these applications, highlighting key areas for future development at the intersection of machine learning, genomic data, and schizophrenia, with the potential to enhance clinical approaches.

## Methods

### Search Strategies

A comprehensive scoping search was conducted to identify recent studies across several electronic databases, including MEDLINE (PubMed), Web of Science, PsycNet (PsycINFO), and Google Scholar, covering the period from 2013 to 2024. The review was conducted using the PRISMA-ScR (Preferred Reporting Items for Systematic Reviews and Meta-Analyses extension for Scoping Reviews) guidelines. The search strategy used both text words and MeSH (Medical Subject Headings) terms, focusing on schizophrenia (eg, “schizophrenia” or “schizophrenic”), genomic data (eg, “genes,” “genetic,” or “genomic”), and machine learning (eg, “artificial intelligence” or “machine learning”). These topics were selected to align with the study’s objectives. Detailed search strategies are provided in Multimedia Appendix 1. The search methodology was developed by the corresponding author, with searches executed by AH and cross-validated by MB. No restrictions were applied regarding setting or geography. The PRISMA checklist is provided in Multimedia Appendix 2.

### Study Eligibility

Studies were included based on the following criteria: (1) the population of interest consisted of patients diagnosed with schizophrenia or the study of schizophrenia, (2) the study used a machine learning approach, and (3) the machine learning model incorporated genomic data features to find specific outcomes. Studies were included regardless of whether they used a single algorithm or multiple algorithms. Excluded from consideration were unpublished literature and studies using artificial intelligence algorithms outside the scope of machine learning. Examples of artificial intelligence algorithms outside the scope of machine learning include search algorithms, expert systems that are not data-driven, and heuristic-based systems. Studies that used machine learning solely to reduce data from genomic datasets were excluded. The search was limited to sources in English and French. Gray literature was not included.

### Data Extraction

Data extraction was performed using a standardized form in Microsoft Excel and was independently counter-verified for consistency and integrity by two authors (AH and MB). Any disagreements regarding the inclusion or exclusion of a study were mutually resolved by the authors. The systematically extracted information included authors, population (sample), primary uses (or intent) of the machine learning algorithms, types of genomic data, types of machine learning algorithm used, main model performances, and key outcomes identified.

### Quality Assessment

The quality of the identified studies was evaluated using the Newcastle-Ottawa Scale for nonrandomized controlled studies and the Cochrane Risk of Bias Tool for randomized controlled trials [31,32]. The Newcastle-Ottawa Scale is a tool used to assess the quality of cohort and case-control studies. It evaluates studies based on three main domains: selection of study groups, comparability of groups, and ascertainment of exposure or outcome [31]. Each domain includes specific criteria, and studies are awarded stars for meeting these criteria, with a maximum of 9 stars indicating the highest quality [31]. The Cochrane Risk of Bias Tool is a comprehensive framework used to assess the risk of bias in randomized controlled trials [32]. It evaluates 7 specific domains: random sequence generation, allocation concealment, blinding of participants and personnel, blinding of outcome assessment, incomplete outcome data, selective reporting, and other potential sources of bias [32]. Each domain is rated as having a low, high, or unclear risk of bias based on predefined criteria [32]. In this scoping review, studies with 1-4 stars on the Newcastle-Ottawa Scale or a high risk of bias by the Cochrane Risk of Bias Tool will be identified as low in quality, 4-6 stars as moderate, and 7-9 stars (or low risk of bias) as high.

## Results

### Description of Studies

**Figure 1:**
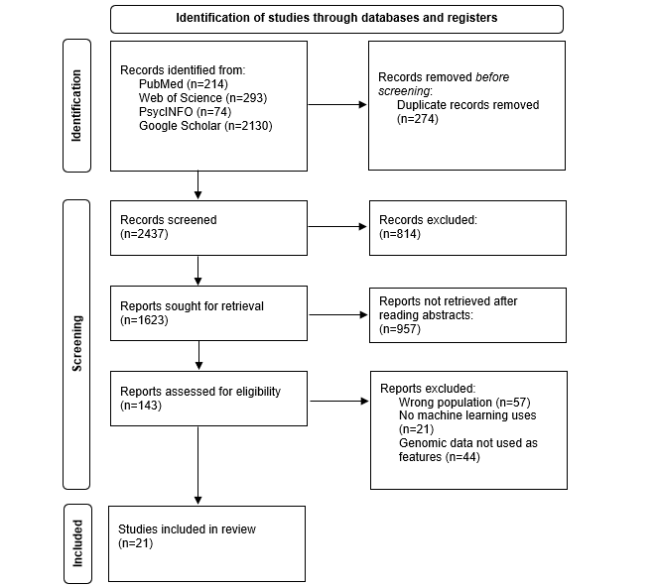
PRISMA (Preferred Reporting Items for Systematic Reviews and Meta-Analyses) flowchart for the inclusion of studies.

The scoping review evaluated studies at the intersection of schizophrenia, genomic data, and machine learning. Initially, the literature search identified 2437 eligible articles after removing duplicates. A total of 814 studies were excluded based on a first analysis of the titles and abstract. Following a second round of abstract screening, 143 full-text articles were thoroughly assessed, with 122 subsequently excluded. This left 21 studies for detailed analysis. A flowchart illustrating the inclusion process is provided in Figure 1, and the specific details of the included studies are available in Multimedia Appendix 3. The studies meeting the inclusion criteria included various algorithms for different tasks. The most common application of machine learning was predicting schizophrenia using genomic data (n=10), followed by identifying features to enhance the understanding of schizophrenia (n=6), drug discovery for patients with schizophrenia (n=2), classifying schizophrenia amongst other mental health disorders (n=2), and predicting the quality of life and global functioning of patients with schizophrenia (n=1).

### Algorithms Used

Several algorithms have been identified in the 21 included studies. The algorithms the most frequently used were support vector machine classifiers (SVM; n=14), random forest (RF) algorithms (n=9), various implementations of neural networks (NN; n=7), and eXtreme Gradient Boosting (XGboost; n=5). Definitions of these popular algorithms are listed below:

- RF: It constitutes an ensemble learning technique. During training, it creates several decision trees and outputs the class, which is the average of the classes of each individual tree [33]. By merging the predictions of several trees, each trained on a different sample of the data, this method increases accuracy and helps avoid overfitting [33].
- SVM: It is an algorithm for supervised machine learning that is applied to regression and classification problems [34]. Finding the ideal hyperplane to divide the data into distinct classes is the fundamental notion behind SVM [34]. Different kernels (a function that quantifies the similarity between a pair of data points) can be used to enhance the performance of the SVM to best fit the data points [35].
- NN: These algorithms are modeled after the composition and operations of the human brain [36]. They are made up of networked layers of nodes, also called neurons, that process and change incoming data to create outputs [36].
- XGboost: It is founded on the gradient boosting principle, which entails building an ensemble of weak learners (usually decision trees) in a stepwise manner [37]. Every new tree seeks to fix the mistakes committed by the ones that came before it [37].

The remaining algorithms can be found in Multimedia Appendix 3.

### Predicting Schizophrenia

Prediction of schizophrenia was identified as the main objective of 10 studies, all of which were deemed of high quality as per the Newcastle-Ottawa Scale ratings. The data used in these studies included differentially expressed genes, polygenic risk scores, genotype and human leukocyte antigen alleles, gene expression microarray data, single nucleotide polymorphisms, long non-coding RNAs, DNA methylation in blood, exomes, and G72 protein levels.

Li et al [38] used differentially expressed gene data from the Gene Expression Omnibus database, applying RF and SVM algorithms, and identified 15 key genes correlated with immune cell infiltration, achieving high diagnostic accuracy for schizophrenia with an area under the curve (AUC) of 0.77 in their test set. Another study, by Bracher-Smith et al [39], used data from the UK Biobank, applied machine learning algorithms such as least absolute shrinkage and selection operator, ridge-penalized logistic regression, SVM, RF, XGboost, NN, and stacked models, and found that while machine learning models incorporating polygenic risk scores and demographic factors showed good discrimination (AUC=0.71), they did not significantly outperform logistic regression in predicting schizophrenia. However, they reported that permutation features importance identified polygenic risk score for schizophrenia (PRS-SZ) as the most important predictor of schizophrenia [39].

Using data from the iPSYCH2012 case cohort, another study integrated genetics and registry data with a deep learning approach to stratify 19,636 patients with schizophrenia with or without major depressive disorder into clinically distinct subgroups characterized by unique disorder severities and comorbidity signatures, with predictive models achieving AUCs of 0.55 to 0.97, and therefore emphasized the importance of data-driven stratification for improving psychiatric diagnosis and prognosis [40]. Similarly, Qi et al [41] analyzed gene expression datasets from untreated schizophrenia patients and controls, identified 14 key gene probes, and used artificial NN to achieve diagnostic accuracy of 91.2% in training and 87.9% in testing and highlighted the potential of machine learning in identifying clinically useful biomarkers for schizophrenia. Another study introduced a sparse deep NN approach for identifying interpretable features for schizophrenia case–control classification using gray matter volume and single nucleotide polymorphism data, demonstrating slightly improved performance over traditional methods and highlighting key brain regions related to schizophrenia [42].

Studies with smaller sample sizes also reported several genomic data-enhanced methodologies to predict schizophrenia. Zhu et al [43] demonstrated that a machine learning model using the expression levels of 6 genes (*GNAI1*, *FYN*, *PRKCA*, *YWHAZ*, *PRKCB*, and *LYN*) in peripheral blood effectively distinguish schizophrenia patients from healthy controls, with the SVM model achieving the highest accuracy (AUC=0.993). Another study also reported the importance of long non-coding RNAs as they provided higher accuracy than coding genes in distinguishing schizophrenia from healthy controls [44].

Also focusing on predicting schizophrenia, a machine learning classifier based on DNA methylation in blood, specifically using correlated regions of systemic interindividual epigenetic variation (CoRSIV) regions and sparse partial least squares regression for discrimination analysis (SPLS-DA), effectively distinguishes schizophrenia patients from controls with a highly positive predictive value (PPV) of 80%, outperforming models based on polygenic risk scores (PRS) [45]. Another machine learning implementation used whole exome sequencing data to identify individuals at high risk for schizophrenia, achieving an accuracy of 85.7% with the XGBoost algorithm and providing further insights into the genetic basis of the disorder [46]. Finally, the last identified study used machine learning algorithms to demonstrate that G72 protein levels alone, without incorporating G72 genetic variations, are effective in distinguishing patients with schizophrenia from healthy controls with high specificity (0.9503) and sensitivity (0.8765) [47].

### Identifying Features of Schizophrenia

A total of 6 included studies aimed at identifying features of schizophrenia or phenotyping using machine learning and genomic data, all of which were assessed as being of high quality. Feng et al [48] identified 6 candidate genes (*SFN*, *KDM5B*, *MYLK*, *IRF3*, *IRF7*, and *ID1*) with diagnostic significance for schizophrenia using machine learning on gene expression data. Another study by Zhu et al [49] attempted to identify immune-related biomarkers in peripheral blood in patients diagnosed with schizophrenia and reported that the mRNA expression of *CLIC3* was significantly decreased in the schizophrenia samples compared with the healthy controls. By using machine learning methods to analyze RNA sequencing data from the dorsolateral prefrontal cortex and amygdala in a postmortem investigation, Liu et al [50] aimed to identify driving biological signals representing schizophrenia. In doing so, they identified 18 genes added to known schizophrenia-associated pathways and expanded the gene network. These results provide a more comprehensive understanding of schizophrenia pathogenesis [50].

De Rosa et al [51] identified biological signals representing schizophrenia in brain tissues of the dorsolateral prefrontal cortex and hippocampus samples from postmortem brains of nonpsychiatric controls and patients with schizophrenia. Using an RF approach, they found 103 additional gene interactions were expanded to schizophrenia-associated networks, which were shared amongst both the dorsolateral prefrontal cortex and amygdala regions [51]. Another study by Feng and Shen [52] used neural networks using programmed cell-death-related genes as features and found 10 candidate hub genes (*DPF2*, *ATG7*, *GSK3A*, *TFDP2*, *ACVR1*, *CX3CR1*, *AP4M1*, *DEPDC5*, *NR4A2*, and *IKBKB*). Finally, a study on fresh frozen postmortem brain tissue aimed to identify DNA methylation patterns specific to patients with schizophrenia.

A cohort of 73 subjects diagnosed with schizophrenia and 52 control samples was analyzed using an unsupervised machine learning approach. As the results were not convincing, the authors reported that, if there are methylation changes associated with schizophrenia, they are diverse, complex, and have a small effect size [53].

### Drug Discovery

A total of 2 studies reported the use of machine learning specifically for drug discovery (or related issues) for patients diagnosed with schizophrenia. Both of them were deemed of high quality. The first study focusing on 2307 patients with schizophrenia from the Chinese Antipsychotics Pharmacogenomics Consortium, 1379 from the Chinese Antipsychotics Pharmacogenetics Consortium, 275 healthy controls used several SVM and RF implementations and identified 6 risk genes for schizophrenia (*LINC01795*, *DDHD2*, *SBNO1*, *KCNG2*, *SEMA7A*, and *RUFY1*), which are involved in cortical morphology and were identified as having genetic-epigenetic interactions linked to treatment response [54]. The other study, by Zhao and So [55], used the expression database ConnectivityMap that contains transcriptomic changes for *HL60*, *PC3*, and *MCF* over several machine learning implementations and reported that the predictive performance of their 5 approaches in cross-validation did not differ substantially, with SVM slightly outperforming the others while stating that repositioning hits are enriched for psychiatric medications considered in clinical trials [55].

### Classifying Schizophrenia Among Other Mental Health Disorders

A total of 2 studies aiming at classifying schizophrenia amongst other mental disorders using machine learning were identified.

The first study by Yang et al [56] aimed at distinguishing schizophrenia from individuals with bipolar disorder, major depressive disorders, and healthy controls. To do so, the authors used differentially expressed genes from 268 individuals (67 patients with schizophrenia, 40 patients with bipolar disorder, 57 patients with major depressive disorders, and 104 healthy controls) over an SVM implementation that achieved an AUC of 0.96 for the schizophrenia group and of 0.71 for the independent set of the classification model. They reported that their model has a strong capacity to classify samples among multiple groups of mental illnesses [56]. Considering the opacity of the implementation, the quality was assessed as moderate for this study.

The other study, by Saardar et al [57], used the dbGaP database (schizophrenia) and the NDAR database (autism spectrum disorder) to compare whole exomes to differentiate between schizophrenia and autism using an XGboost model. They achieved an average validation accuracy of over 5 folds was 88% for both the single nucleotide variants-based model and gene-based model and reported that the ion transmembrane transport, neurotransmitter transport, and microtubule or cytoskeleton processes were of importance for schizophrenia [57]. The quality of this study was determined to be high based on our assessment.

### Predicting Quality-of-Life and Global Functioning

Only one of the included studies focused on predicting the quality of life and global functioning of patients diagnosed with schizophrenia. This study was of high quality as per the quality assessment. Using data from 302 patients with schizophrenia in the Taiwanese population, Lin et al [58] compared a bagged ensemble of several machine learning algorithms to different permutations of these algorithms to predict functional outcomes of patients with schizophrenia. Their analysis revealed that the bagging ensemble algorithm with feature selection outperformed other predictive algorithms in forecasting the quality-of-life functional outcome of schizophrenia using the G72 rs2391191 and MET rs2237717 SNPs [58].

## Discussion

### Principal Results

This scoping review aimed to identify the different ways machine learning algorithms can be applied to genomic data in the study of schizophrenia. A total of 21 studies were fully analyzed, and 5 uses of machine learning algorithms on genomic data were identified: predicting schizophrenia, identifying features of schizophrenia, drug discovery, classifying schizophrenia amongst other mental health disorders, and predicting quality-of-life and global functioning. The studies were overall of high quality.

### Comparison With Previous Work

The application of predictive models to forecast mental health disorders, such as schizophrenia, is gaining importance in medical research [59]. These models hold the potential to significantly assist clinicians in patient evaluation, particularly given the heterogeneity inherent to schizophrenia [60]. However, as observed in the identified studies, these models vary greatly in their implementation with diverging accuracy and validation methodologies. It is important to consider the implementation of these models as well as their accuracy and the techniques used to cross-validate the model, especially when using genomic data, as this could hinder their external validity [61]. The results found in the identified studies reinforce the premise that the genetic architecture of schizophrenia has proven to be very complex, heterogeneous, and polygenic and that a vast array of features could be integrated to improve predictive models [62]. Similarly, finding genomic-related risk factors of schizophrenia in such a model could help in distinguishing between this disease and other mental disorders, which may explain why classifying schizophrenia among other mental health disorders was one of the identified uses.

It is unsurprising that machine learning has been used to identify features of schizophrenia, as this has been done in other medical fields. Using candidate genes, it can be possible for clinicians to better understand common diseases and complex traits [63]. In psychiatry, psychiatric genomics is a rapidly advancing field that shows great promise for enhancing risk prediction, prevention, diagnosis, treatment selection, and the understanding of the pathogenesis of patients’ symptoms [64]. As an example, some genes and functional genomic data linked to complex features of schizophrenia demonstrated that specific alleles may confer risk to the disorder by directly affecting synaptic function in adulthood [65].

As for drug discovery, literature reviews on the subject support that machine learning techniques can improve decision-making in pharmaceutical data across various applications [66,67]. It is also reported that combining machine learning techniques with genomic data has the potential to speed up the process and reduce failure rates in drug discovery and development [67]. This may explain why two studies focused specifically on schizophrenia in the context of drug discovery were identified. There is an increasing effort to develop pharmaceutical treatments, given the 20%-30% rate of treatment resistance observed in patients with this disorder [4].

Finally, quality-of-life assessment and functioning of patients with schizophrenia is trending in this field, which may explain why this use was identified in one study [68,69]. Another recent study on quality of life and genome-wide analyses of quality of life in psychosis, which used linear regression on 3684 participants (including 1119 psychosis patients), reported that numerous clinical and genetic associations with quality of life can be used in the daily care of these patients and enhance their overall well-being. These findings support the idea that more work should be conducted in this area in the future [70].

In the future, the information gathered by the use of machine learning in this area may provide the basis for more research projects. Through the identification of current knowledge gaps, scientists can narrow their attention and investigate novel genetic and biological markers that may have escaped their notice in the past in the development of machine learning models. This information may pave the way for the development of innovative therapeutic approaches, individualized treatment programs, and a better comprehension of the fundamental pathology of schizophrenia. To effectively handle the intricate problems presented by schizophrenia, machine learning techniques might need to be integrated with genomic data as they develop, and the genes identified in this review might help researchers select key features to enhance their mathematical models. This addition might lead to advancements in both basic science and therapeutic applications.

### Limitations of This Study

This scoping review highlighted the various applications of machine learning algorithms using genomic data in the field of schizophrenia. Despite the relevance of this recension, it has a few limitations. The heterogeneity of diagnostic criteria for schizophrenia is a significant concern, as it is not addressed in half of the studies reviewed. Furthermore, the limited number of studies identified indicates the novelty of this field, necessitating future reviews to confirm findings. There is also a lack of external validation in samples differing from the training sample, such as those from different nationalities, raising questions about the generalizability of the results. Notably, no studies have concretely tested these algorithms in clinical settings, particularly for the prediction of schizophrenia, which remains an unmet need in the research. Due to the heterogeneity of the identified studies and the varying metrics used to assess precision and validate the machine learning models, performance comparisons were not conducted. Furthermore, studies on generic models using genomic data to predict overall mental health, rather than specifically focusing on schizophrenia, were excluded, as well as unpublished literature. This may have led to the omission of a small portion of relevant studies.

### Conclusions

Considering the heterogeneity of clinical presentations observed in schizophrenia, genomic data combined with machine learning algorithms have been implemented to address several facets of this disorder. From the 21 studies analyzed, 5 main uses were identified: predicting schizophrenia, identifying schizophrenia features, discovering drugs, classifying schizophrenia amongst other mental health disorders, and predicting the quality of life of patients. These uses have potential implications as they could assist clinicians in providing a more personalized approach to their patients diagnosed with schizophrenia, considering the complexity of this diagnosis. There is still a limited amount of literature on the subject, and this study provides a first overview of machine learning applications of genomic data for schizophrenia. Future research is essential to further evaluate the portability of the models identified and their potential clinical applications.

## Appendix

Multimedia Appendix 1 Electronic search strategy for the scoping review conducted.

Multimedia Appendix 2 PRISMA-ScR (Preferred Reporting Items for Systematic Reviews and Meta-Analyses extension for Scoping Reviews) checklist.

Multimedia Appendix 3 Systematic review study selection detailed results.

## Abbreviations

AUC: area under the curve
CNV: copy number variation
CoRSIV: correlated regions of systemic interindividual epigenetic variation
GWAS: genome-wide association studies
MeSH: Medical Subject Headings
NN: neural networks
PPV: positive predictive value
PRISMA-ScR: Preferred Reporting Items for Systematic Reviews and Meta-Analyses extension for Scoping Reviews
PRS: polygenic risk scores
PRS-SZ: polygenic risk score for schizophrenia
RF: random forest
SNP: single nucleotide polymorphism
SPLS-DA: sparse partial least squares regression for discrimination analysis
SVM: support vector machine
XGboost: eXtreme Gradient Boosting
