# Supplementary material for: Exploring the Intersection of Schizophrenia, Machine Learning, and Genomics: Scoping Review
Source: JMIR Bioinform Biotechnol. 2024 Nov 15;5:e62752. doi: 10.2196/62752 (PMC11607571; doi:10.2196/62752)
Supplement: Multimedia Appendix 1 [file bioinform_v5i1e62752_app1.docx]

**Supplementary Online Content**

Hudon, A., Beaudoin, M, Phraxayavong, K., Potvin, S., Dumais, A. Exploring the Intersection of Schizophrenia, Machine Learning, and Genomics: A Scoping Review

**Multimedia Appendix 1.** Electronic search strategy for the scoping review conducted.

**Multimedia Appendix 1. Electronic search strategy for the scoping review conducted.**

| **Database; Search** | **Search Terms** |
| --- | --- |
|  |  |
| PubMed; k= 214 | ((Schizophrenia[Title/Abstract] OR Schizophrenic[Title/Abstract] OR Schizophrenia"[MeSH]) AND ("Machine Learning"[Title/Abstract] OR "Artificial Intelligence"[Title/Abstract] OR AI[Title/Abstract] OR "Deep Learning"[Title/Abstract] OR "Neural Networks"[Title/Abstract] OR "Machine Learning"[MeSH]) AND (Gene[Title/Abstract] OR Genetic[Title/Abstract] OR Genetics[Title/Abstract] OR Genomic[Title/Abstract] OR Genome[Title/Abstract] OR "Genes"[MeSH])) |
| Web of Science; k= 293 | TS=(Schizophrenia OR Schizophrenic) AND  TS=("Machine Learning" OR "Artificial Intelligence" OR AI OR "Deep Learning" OR "Neural Networks") AND  TS=(Gene OR Genetic OR Genetics OR Genomic OR Genome) |
| PsycInfo; k = 74 | ((TI(Schizophrenia) OR AB(Schizophrenia) OR TI(Schizophrenic) OR AB(Schizophrenic)) AND  (TI("Machine Learning") OR AB("Machine Learning") OR TI("Artificial Intelligence") OR AB("Artificial Intelligence") OR TI(AI) OR AB(AI) OR TI("Deep Learning") OR AB("Deep Learning") OR TI("Neural Networks") OR AB("Neural Networks")) AND  (TI(Gene) OR AB(Gene) OR TI(Genetic) OR AB(Genetic) OR TI(Genetics) OR AB(Genetics) OR TI(Genomic) OR AB(Genomic) OR TI(Genome) OR AB(Genome))) |
| Google Scholar; k= 2130 | (("Schizophrenia" OR "Schizophrenic") AND ("Machine Learning" OR "Artificial Intelligence" OR "AI" OR "Deep Learning" OR "Neural Networks") AND ("Gene" OR "Genetic" OR "Genetics" OR "Genomic" OR "Genome") AND ("Biomarkers" OR "Diagnosis" OR "Prediction") AND ("RNA-Seq" OR "SNP" OR "Epigenetics") AND ("Support Vector Machine" OR "Random Forest" OR "XGBoost") AND ("Functional Outcome" OR "Clinical Outcome" OR "Treatment Response") AND ("Cross-Validation" OR "Feature Selection" OR "Accuracy" OR "AUC")) |
