# Supplementary material for: Exploring the Intersection of Schizophrenia, Machine Learning, and Genomics: Scoping Review
Source: JMIR Bioinform Biotechnol. 2024 Nov 15;5:e62752. doi: 10.2196/62752 (PMC11607571; doi:10.2196/62752)
Supplement: Multimedia Appendix 3 [file bioinform_v5i1e62752_app3.docx]

**Supplementary Online Content**

Hudon, A., Beaudoin, M, Phraxayavong, K., Potvin, S., Dumais, A. Exploring the Intersection of Schizophrenia, Machine Learning, and Genomics: A Scoping Review

**Multimedia Appendix 3.** Systematic review study selection detailed results.

This supplementary material has been provided by the authors to give readers additional information about their work.

**Table S3. Systematic review study selection detailed results.**

| **Studies** | **Population** | **Uses** | **Genomic Data Types** | **Algorithms used** | **Main Model Performance** | **Main outcome** | **Quality Assessment** |
| --- | --- | --- | --- | --- | --- | --- | --- |
| Li et al., 2022 [38] | Gene Expression Omnibus database (post-mortem human brains, N not specified) | Predicting schizophrenia | Differentially expressed genes | RF, SVM | AUC of RF: 0.83 (0.77-0.89), AUC of SVM: 0.81 (0.74-0.88) | 15 key genes (SLC1A3, AQP4, GJA1, ALDH1L1, SOX9, SLC4A4, EGR1, NOTCH2, PVALB, ID4, ABCG2, METTL7A, ARC, F3 and EMX2) involved in schizophrenia | High |
| Bracher-Smith et al., 2022 [39] | UK Biobank (blood samples from 738 patients and 3,690 randomly sampled controls, all self-reported as white British or Irish) | Predicting schizophrenia | Polygenic risk scores | LASSO and ridge-penalised logistic regression, LSVM, SVM-RBF, RF, XGBoost, NN and stacked models | Models combining all predictors showed the highest discrimination (LSVM, AUROC = 0.71) | Permutation feature importance identified PRS-SZ as the most important predictor | High |
| Allesøe et al., 2022 [40] | iPSYCH2012 case cohort sample (dry blood spots taken at birth for 19,636 patients – 3,896 of which were diagnosed with schizophrenia - and 22,467 population controls) | Predicting schizophrenia | Genotype and human leukocyte antigen alleles, Polygenic risk scores | DL model | Schizophrenia clusters with AUCs ranging from 0.71 to 0.86 | No significant signal for individual SNPs in the genetics data and only a few SNPs with significantly different homozygote/heterozygote distributions. | High |
| Qi et al., 2022 [41] | 201 patients and 278 healthy controls (post-mortem, from dbGaP study phs0 0 0979.v1.p1) | Predicting schizophrenia | Gene expression microarray data. | XGBoost | AUC on 10-fold cross-validation of 0.76 and an AUC of 0.76 on testing data, not used during training | Above-average performance from machine learning classification of schizophrenia cases and controls using brain gene expression microarray data. | High |
| Chen et al., 2021 [42] | 1,443 patients from multiple studies (MCIC, COBRE, FBIRN, NU, BSNIP, TOPm and HUBIN) | Predicting schizophrenia | Single nucleotidepolymorphism | DNN, ICA+SVM | DNN slightly outperformed independent component analysis with an average error rate of 28.98% on external data. | An interpretable, sparse DNN approach was designed to allow identifying, refining and interpreting features used in classification. | High |
| Zhu et al., 2021 [43] | 43 patients and 50 healthy controls (peripheral blood) | Predicting schizophrenia | Gene expression microarray data. | ANN, XGBoost, SVM, DT, RF | AUC of SVM: 0.993, sensitivity = 1.000, specificity = 0.895, and Youden index = 0.895 | The SVM model with six factors (GNAI1, FYN, PRKCA, YWHAZ, PRKCB, and LYN genes) was the best model for distinguishing patients with schizophrenia from healthy individuals | High |
| Liu et al., 2021 [44] | 254 patients from the CommonMind consortium, all from European ancestry (dorsolateral prefrontal cortex RNA-seq data) | Predicting schizophrenia | Differentially expressed genes, long non-coding RNAs | RF | Mean predictive accuracy for schizophrenia patients was 67% based on coding genes, and 96% based on long non-coding RNAs. | The long non-coding RNAs more accurately reflect the characteristics of patients with schizophrenia tissue than mRNA because they regulate gene expression at multiple levels, not just at the mRNA level. | High |
| Gunasekara et al., 2021 [45] | 414 patients and 433 non-psychiatric controls from Illumina HM450 datasets (five cohorts used from model training), all self-identified as born in British Isles | Predicting schizophrenia | DNA methylation in blood | Sparse partial least squares discriminate analysis | The CoRSIV-based model classified 303 individuals as cases with a positive predictive value of 80%, far surpassing the performance of a model based on polygenic risk score. | The results indicate two innate dimensions of schizophrenia risk: one based on genetic, and the other on systemic epigenetic variants. | High |
| Trakadis et al., 2019 [46] | 2,545 patients + 2,545 healthy controls randomly selected from the Swedish population registry. Data extracted from the from the dbGaP study phs000473.v1.p1. For all participants, both parents had to be born in Scandinavia. | Predicting schizophrenia | Exomes | XGBoost | The p-value of 9.11 x 10^-179 indicates that the XGBoost algorithm significantly outperforms a random predictor that simply predicts the majority class. XGBoost achieved the highest performance metrics, with a specificity of 86.6%, sensitivity of 84.9%, precision of 86.9%, and recall of 84.9%. | This study implemented an algorithm to predict patients at high risk for schizophrenia based on rare, predicted functional, variants. | High |
| Lin et al., 2018 [47] | 89 patients and 60 healthy controls, all Han Chinese, physically and neurogically healthy, non-smokers, no substance abuse or dependence | Predicting schizophrenia | Single nucleotide polymorphisms and G72 protein levels | Logistic regression, Naive Bayes, and C4.5 decision tree | Naive Bayes model: sensitivity = 0.7969, specificity = 0.9372, AUC = 0.9356 ; Naive Bayes model with G72 protein alone specificity: 0.9503 | This study suggests that the G72 protein alone, without including the two G72 SNPs, might be sufficient to identify schizophrenia patients. | High |
| Feng et al., 2023 [48] | Gene Expression Omnibus - Schizophrenia dataset GSE92538 for training (58 patient samples, 175 control samples) and GSE21935 for testing (23 patient samples, 19 control samples) | Identifying features of schizophrenia | Differentially expressed genes | RF | AUC for nomograph of candidate genes: AUC 0.83, CI 0.97–0.69 | Six potential candidate genes (SFN, KDM5B, MYLK, IRF3, IRF7, and ID1) have been identified. | High |
| Zhu et al., 2023 [49] | 158 patients and 151 healthy controls from four datasets: GSE18312 (PBMC), GSE165604 (blood lymphocytes), GSE27383 (PBMC), GSE 38484 (whole blood) | Identifying features of schizophrenia | Differentially expressed genes | RF, LASSO, SVM-RFE | AUC of CLIC3 was 0.69 | The mRNA expression of CLIC3 was significantly decreased in the schizophrenia samples compared to the healthy controls. | High |
| Liu et al., 2022 [50] | DLPFC tissues from 120 patients and 134 healthy controls + post-mortem amygdala samples from 22 patients and 24 healthy samples - all from European ancestry | Identifying features of schizophrenia | Differentially expressed genes | RF | N/A | 103 additional gene interactions were expanded to schizophrenia-associated networks, which were shared among both the DLPFC and amygdala regions. | High |
| De Rosa et al., 2022 [51] | DLPFC and hippocampus samples from post-mortem brains of 20 patients and 20 non-psychiatric controls, obtained from The Human Brain and Spinal Fluid Resource Center (Los Angeles, USA) | Identifying features of schizophrenia | mRNA and protein levels of pre- and post-synaptic key molecules involved in the glutamatergic synapse functioning | iRF | DLPFC: iRF achieved a relatively small prediction error (Brier Score = 0.186) and a very high discriminatory power (AUC = 0.80, 95% CI: 0.65–0.92); hippocampu: iRF achieved a smaller prediction error (Brier Score = 0.165) and a higher discriminatory power (AUC = 0.85, 95% CI: 0.71–0.95). | This study confirms the significant role of the glutamatergic synapse in the molecular pathophysiology of schizophrenia. Additionally, molecular markers related to the glutamate synapse were able to effectively distinguish between schizophrenia patients and healthy individuals. | High |
| Feng & Shen, 2023 [52] | Gene Expression Omnibus database, schizophrenia datasets (GSE92538 and GSE21935) | Identifying features of schizophrenia | Programmed cell-death related genes | ANN, CC | AUC of training set : 0.91, CI 0.95–0.86, AUC of validation set: AUC 0.94, CI 1.00–0.85 | 10 candidate hub genes (DPF2, ATG7, GSK3A, TFDP2, ACVR1, CX3CR1, AP4M1, DEPDC5, NR4A2, and IKBKB) identified. | High |
| Torabi Moghadam et al., 2019 [53] | Fresh frozen post-mortem brain tissue from a cohort of 73 patients and 52 controls obtained from different international brain banks | Identifying features of schizophrenia | Differentially expressed genes | Unsupervised learning method : methylSaguaro | Accuracy of the model: 60% | If there are methylation changes associated with schizophrenia, they are diverse, complex, and have a small effect size. | High |
| Guo et al., 2023 [54] | 2307 patients from the CAPOC trial (discovery cohort), 1379 patients from the CAPEC trial (external validation cohort), and 275 healthy controls from the local community. All participants were of Han Chinese ancestry and right-handed. | Drug discovery | Genotype information, Polygenic risk scores , DNA methylation profiles, Polymethylation scores, Epigenetic clocks, Differentially methylated regions , Methylation quantitative trait loci , Chromatin interaction data | RF, QRF, SVMPoly, SVM-RBF | Discovery cohort: AUC=0.874 (95% CI 0.867–0.881), R2=0.478, r=0.76 (95% CI 0.74–0.78); External validation cohort: AUC=0.851 (95% CI 0.841–0.861), R2=0.507, r=0.75 (95% CI 0.72–0.77) | Six risk genes for schizophrenia (LINC01795, DDHD2, SBNO1, KCNG2, SEMA7A, and RUFY1), which are involved in cortical morphology, were identified as having genetic-epigenetic interactions linked to treatment response. | High |
| Zhao & So., 2018 [55] | Drug expression data from the Connectivity Map, which captured transcriptomic changes when three cell lines were treated with a drug or chemical | Drug discovery | Transcriptomic changes for HL60, PC3, MCF7 | DNN, SVM, ElasticNet, RF, XGBoost | The predictive performance of the five approaches in cross validation did not differ substantially, with SVM slightly outperforming the others. | Repositioning hits are enriched for psychiatric medications considered in clinical trials. | High |
| Yang et al., 2022 [56] | 268 patients (67 with schizophrenia, 40 with bipolar disorder, 57 with major depressive disorders) and 104 healthy controls. All prefrontal cortex samples from three microarray datasets | Classifying schizophrenia amongst other mental health disorders | Differentially expressed genes | SVM | AUC for schizophrenia group: 0.96, Independent set for classification model : 0.71 | A strong capacity to classify samples among multiple groups was demonstrated. | Moderate |
| Saardar et al., 2020 [57] | Schizophrenia: 591 trios from DdbGaP dataset (phs000687.v1vp1), collected in Bulgaria. Autism spectrum disorder: 2392 trios from NDAR | Classifying schizophrenia amongst other mental health disorders | Whole exome | XGBoost | Average validation accuracy over five folds was 88% for both the SNV-based model and gene-based model (to discriminate schizophrenia from autism spectrum disorder). | Ion transmembrane transport, neurotransmitter transport, and microtubule/cytoskeleton processes were highlighted for schizophrenia. | High |
| Lin et al., 2021 [58] | Venous blood from 302 patients recruited in Taiwan | Predicting quality-of-life and global functioning | Single nucleotide polymorphisms | Bagging ensemble (SVM, MFNN, Linear regression, RF) | RMSE for bagging ensemble with feature selection for quality of life: 8.6766 ± 1.0421, RMSE for same model for Global assessment of functioning: 9.6982 ± 1.3354 | The analysis revealed that the bagging ensemble algorithm with feature selection outperformed other predictive algorithms in forecasting the QLS functional outcome of schizophrenia using the G72 rs2391191 and MET rs2237717 SNPs. | High |

*Abbreviations: ANN: Artificial Neural Network, AUC: Area Under the Curve, AUROC: Area Under the Receiver Operating Characteristic curve, CAPOC:* *Chinese Antipsychotics Pharmacogenomics Consortium, CAPEC:* *Chinese Antipsychotics Pharmacogenetics Consortium, CC: Consensus Clustering, CI: Confidence interval, DLPFC: Dorsolateral prefrontal cortex, DNN: Deep Neural Network, DL model: Deep Learning Model, DT: Decision Tree, ElasticNet: Elastic Net Regularization, iRF: Iterative Random Forest, LASSO: Least Absolute Shrinkage and Selection Operator, LSVM: Linear Support Vector Machine, MFNN: Multi-Layer Feedforward Neural Network, NN: Neural Networks, QRF: Quantile Regression Forests, RF: Random Forest, RMSE: Root Mean Square Error, SNPs:* *Single Nucleotide Polymorphisms SVM: Support Vector Machine, SVM-RBF: Support Vector Machine with Radial Basis Function kernel, SVM-RFE: Support Vector Machine with Recursive Feature Elimination, , XGBoost: eXtreme Gradient Boosting.*
